# Bicarbonate transport of airway surface epithelia in luminally perfused mice bronchioles

**DOI:** 10.1186/s12576-022-00828-2

**Published:** 2022-02-23

**Authors:** Libin Liu, Akiko Yamamoto, Makoto Yamaguchi, Itsuka Taniguchi, Nao Nomura, Miyuki Nakakuki, Yuka Kozawa, Tomoya Fukuyasu, Mayuko Higuchi, Erina Niwa, Tsutomu Tamada, Hiroshi Ishiguro

**Affiliations:** 1 Department of Human Nutrition, Nagoya University Graduate School of Medicine, Nagoya, Japan; 2 Department of Respiratory Medicine, Tohoku University Graduate School of Medicine, Sendai, Japan; 3 Research Center of Health, Physical Fitness, and Sports, Nagoya University, Furo-cho E5-2 (130), Chikusa-ku, Nagoya, 464-8601 Japan

**Keywords:** Distal airway, HCO_3_^−^ secretion, Bronchiole, Microperfusion, Surface epithelial cells, Intracellular pH

## Abstract

HCO_3_^−^ secretion in distal airways is critical for airway mucosal defense. HCO_3_^−^/H^+^ transport across the apical membrane of airway surface epithelial cells was studied by measuring intracellular pH in luminally microperfused freshly dissected mice bronchioles. Functional studies demonstrated that CFTR, ENaC, Cl^−^–HCO_3_^−^ exchange, Na^+^-H^+^ exchange, and Na^+^–HCO_3_^−^ cotransport are involved in apical HCO_3_^−^/H^+^ transport. RT-PCR of isolated bronchioles detected fragments from Cftr, α, β, γ subunits of ENaC, Ae2, Ae3, NBCe1, NBCe2, NBCn1, NDCBE, NBCn2, Nhe1, Nhe2, Nhe4, Nhe5, Slc26a4, Slc26a6, and Slc26a9. We assume that continuous decline of intracellular pH following alkaline load demonstrates time course of HCO_3_^−^ secretion into the lumen which is perfused with a HCO_3_^−^-free solution. Forskolin-stimulated HCO_3_^−^ secretion was substantially inhibited by luminal application of CFTR_inh_-172 (5 μM), H_2_DIDS (200 μM), and amiloride (1 μM). In bronchioles from a cystic fibrosis mouse model, basal and acetylcholine-stimulated HCO_3_^−^ secretion was substantially impaired, but forskolin transiently accelerated HCO_3_^−^ secretion of which the magnitude was comparable to wild-type bronchioles. In conclusion, we have characterized apical HCO_3_^−^/H^+^ transport in native bronchioles. We have demonstrated that cAMP-mediated and Ca^2+^-mediated pathways are involved in HCO_3_^−^ secretion and that apical HCO_3_^−^ secretion is largely mediated by CFTR and H_2_DIDS-sensitive Cl^−^–HCO_3_^−^ exchanger, most likely Slc26a9. The impairment of HCO_3_^−^ secretion in bronchioles from a cystic fibrosis mouse model may be related to the pathogenesis of early lung disease in cystic fibrosis.

## Introduction

The airway surface liquid (ASL) is a thin layer of fluid covering the luminal surface of airway epithelium. The ASL is composed of inner periciliary liquid layer (PCL) and outer single-layer thin mucus. Proper volume/depth, viscosity, and pH of ASL are required for efficient mucociliary clearance and antimicrobial activity [11, 38, 47].

It is widely accepted that the volume/depth of PCL is determined by Cl^−^ secretion via cystic fibrosis transmembrane conductance regulator (CFTR) and Ca^2+^-activated Cl^−^ channel (CaCC) and Na^+^ absorption via epithelial Na^+^ channel (ENaC) [30, 33]. In proximal airways, Cl^−^ secretion is mostly derived from serous cells of submucosal glands [5, 8, 17]. In distal airways, submucosal glands are absent [10, 35] and concurrent Cl^−^ secretion and Na^+^ absorption was observed in surface epithelial cells [44]. Loss of CFTR function due to severe pathogenic variants in both alleles of the *CFTR* gene causes cystic fibrosis (CF). The initial event of CF lung disease is characterized by low PCL volume, which is thought to be achieved by defective CFTR-mediated Cl^−^ secretion and abnormally elevated Na^+^ absorption via ENaC [33].

Evidence has accumulated to indicate that HCO_3_^−^ transport is important in airway mucosal defense. HCO_3_^−^ concentration affects physical properties of mucus [4, 39] and mucociliary transport in ex vivo pig trachea under acetylcholine (ACh) stimulation was more dependent on HCO_3_^−^ secretion than Cl^−^ secretion [13]. ASL pH in vivo newborn CF pigs was more acidic compared to wild-type and the impaired bacterial-killing activity of CF ASL was rescued by adding NaHCO_3_ [38]. Cellular mechanisms for HCO_3_^−^ transport in airways have been studied using cultured human nasal epithelial cells [36, 37] and Calu-3 cells, a model of serous cells of submucosal glands [20, 25, 28]. However, HCO_3_^−^ transport in distal airways/bronchioles is not well understood. ASL pH was more alkaline in lower airways than in upper airways in human [34]. Thus, a balance of HCO_3_^−^ and H^+^ secretion may shift to HCO_3_^−^ secretion in distal airways.

Distal airways contribute to 85–90% of the total epithelial surface area of conducting airways [10, 50]. Moreover, mucus plugging and obstruction of bronchioles are among the earliest events of CF lung disease, suggesting that regulation of epithelial ion transport in distal airways is critical for normal lung physiology [46]. However, the assessment of ion transport in distal airways/bronchioles has been limited because of the small size, complex anatomy and relative inaccessibility of structures [10]. Measurement of transmembrane potential in sheep, porcine, and human bronchioles identified Na^+^ and Cl^−^ conductive pathways [2, 6, 9]. Aquaporin-mediated transepithelial water permeability was identified in guinea pig bronchioles [18]. Measurement of transepithelial potentials by a capillary-Ussing chamber revealed concurrent fluid secretion and absorption and HCO_3_^−^ secretion in human bronchioles [45]. However, characteristics and cellular mechanisms of HCO_3_^−^ transport in distal airways/bronchioles have not been fully investigated.

In the present study, HCO_3_^−^ transport in surface epithelial cells of native bronchioles was studied by measuring intracellular pH (pH_i_) in luminally microperfused freshly dissected mice bronchioles. HCO_3_^−^ transport in bronchioles from a CF mouse model was also studied.

## Methods

### Ethics approval

The study was approved by the Ethical Committee of Nagoya University on Animal Use for Experiment (approval No. M210457-003) and the Recombinant DNA Experiment Safety Committee of Nagoya University (approval No. 20-93).

### Isolation of bronchioles from mice lung

A CF mouse model in which the F508del mutation was introduced in the mouse *Cftr* with the C57BL/6J genetic background (ΔF mouse) [53] was purchased from the Jackson Laboratory (Bar Harbor, ME). ΔF mice and their wild-type littermates were bled in Center for Research of Laboratory Animals and Medical Research Engineering, Nagoya University. Mice (8–10 weeks of age) of either sex were suffocated with CO_2_. The thorax was opened and the ice-cold standard HCO_3_^−^-buffered solution was gently injected into the trachea to fill the lungs. The lungs were then removed and the segments of conducting bronchioles (the third or fourth branches, 150–180 μm in inner diameter) were micro-dissected using sharpened needles in the ice-cold standard HCO_3_^−^-buffered solution.

### Solutions

The standard HCO_3_^−^-buffered solution contained (mM): 115 NaCl, 5 KCl, 1 CaCl_2_, 1 MgCl_2_, 10 d-glucose, and 25 mM NaHCO_3_, and was equilibrated with 95% O_2_–5% CO_2_. The 25 mM HCO_3_^−^–0% CO_2_ solution was gassed with 100% O_2_ (pH: ~ 7.8) and thus was nominally free of CO_2_. The standard Hepes-buffered solution contained (mM): 130 NaCl, 5 KCl, 1 CaCl_2_, 1 MgCl_2_, 10 d-glucose, and 10 Na-Hepes, and was equilibrated with 100% O_2_. The Cl^−^-free HCO_3_^−^-buffered solution contained (mM): 115 Na-gluconate, 2.5 K_2_HPO_4_, 1 CaSO_4_, 1 MgSO_4_, 10 d-glucose, and 25 mM NaHCO_3_, and was equilibrated with 95% O_2_–5% CO_2_. The Cl^−^-free Hepes-buffered solution contained (mM): 130 Na-gluconate, 2.5 K_2_HPO_4_, 1 CaSO_4_, 1 MgSO_4_, 10 d-glucose, and 10 Na-Hepes, and was equilibrated with 100% O_2_. The Na^+^-free HCO_3_^−^-buffered solution contained *N*-methyl-d-glucamine (NMDG) in place of NaCl, choline bicarbonate in place of NaHCO_3_, and 10 μM atropine to prevent the possible activation of muscarinic receptors by choline. The Na^+^-free HEPES-buffered solution contained NMDG-Cl in place of NaCl, and Hepes-acid in place of Na-Hepes. In the HCO_3_^−^-buffered solution containing 20 mM NH_4_Cl, the concentration of NaCl was reduced to maintain osmolarity. All solutions, except for the 25 mM HCO_3_^−^–0% CO_2_ solution, were adjusted to pH 7.4 at 37 ℃.

### Microperfusion of isolated bronchioles

**Fig. 1:**
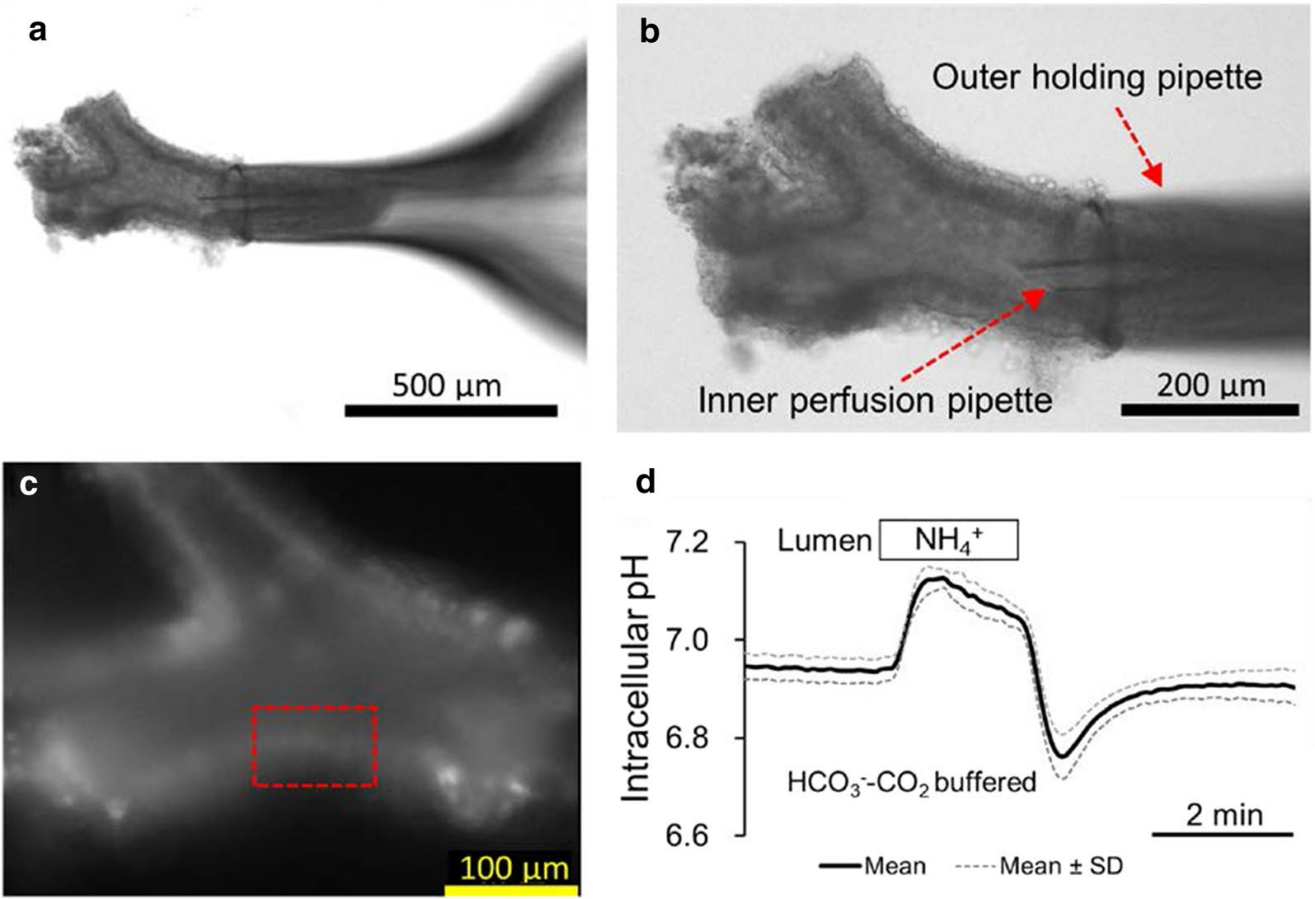
Luminal microperfusion of an isolated bronchiole and measurement of intracellular pH. **a**, **b** The proximal end of an isolated bronchiole (inner diameter: ~ 150 μm) is held and cannulated for luminal microperfusion (20–30 μl/min). The representative bronchiole is bifurcated. The luminal perfusate leaving the distal end of the bronchiole is diluted and washed away by the flow (~ 3 ml/min) of solution through the bath. **c** Fluorescence of BCECF in surface epithelial cells lining the bronchiole. Small regions of the surface epithelium are selected (such as a rectangle) and the intracellular pH (pH_i_) is estimated by microfluorometry at 37 °C. **d** Isolated bronchioles were bilaterally perfused with the standard HCO_3_^−^-buffered solution and NH_4_Cl (20 mM) was applied to the lumen. Time course changes of pH_i_ are shown as means ± SD of 5 experiments

The lumen of the isolated bronchiole segments was microperfused by applying a method to microperfuse isolated pancreatic ducts [22]. One end of bronchiole was cannulated for luminal microperfusion (Fig. 1a and b). The concentric pipette arrangement consisted of an outer holding pipette, an inner perfusion pipette, and a silica inner capillary for exchange of solutions. The combination of inner silica capillary and waste line enables rapid exchange of luminal solutions. The lumen was perfused at 20–30 μl/min while the bath was perfused at ~ 3 ml/min and maintained at 37 ℃. The luminal perfusate leaving the other end of the bronchiole was diluted and washed away by the much greater flow of solution through the bath, which prevented the luminal perfusate from gaining access to the basal surface of the bronchiole.

### Measurement of intracellular pH of bronchiole surface epithelium

The intracellular pH (pH_i_) in the epithelial cells was estimated by microfluorometry using the pH-sensitive fluoroprobe 2',7'-bis-(2-carboxyethyl)-5(6)-carboxyfluorescein (BCECF). After cannulating a bronchiole for luminal microperfusion, the epithelial cells were loaded with BCECF by perfusing the lumen with a solution containing the acetoxymethyl ester BCECF-AM (5 μM) for 10 min. Small regions of the bronchiole surface epithelium (Fig. 1c) were illuminated alternately at 430 and 480 nm and fluorescence was measured at 530 nm (F_430_ and F_480_). Values of pH_i_ were calculated from the F_480_/F_430_ ratio after correction for the endogenous tissue fluorescence measured prior to loading with BCECF. Calibration data were obtained by the high K^+^-nigericin technique [36, 48].

### Reverse transcriptase-polymerase chain reaction

Messenger RNA expression of several ion transporters and channels was examined in isolated bronchioles and tracheal mucosa by polymerase chain reaction (PCR) (Table 1). Primers were derived from published sequences with GenBank accession numbers. The PCR protocol was: 96 ℃, 25 s; 60 ℃, 30 s; 72 ℃, 40 s; 35 cycles. Templates for positive controls were complementary DNAs (cDNAs) prepared from lung, kidney, heart, colonic mucosa, brain and stomach mucosa. Glyceraldehyde-3-phosphate dehydrogenase (GAPDH)-specific primers (452 bp) were used for the positive controls.

**Table 1:** Primer pairs used to amplify ion transporters and channels

Name	Accession number	Primer	Sequence (5′ → 3′)	Size (bp)
Cftr	NM_021050.2	Forward	(4070) atggaaagttgcagatgaggtt	399
		Reverse	(4468) ctcatcttttccgaggagctaa	
Slc26a3 (Dra)	NM_021353.3	Forward	(556) cctactttttcttgggcacatc	385
		Reverse	(940) accgactccaggactttgaata	
Slc26a4 (Pendrin)	NM_011867.4	Forward	(974) cggcatcctctccattatctac	465
		Reverse	(1438) gccacaaaacaggagaaaaatc	
Slc26a6 (Pat1)	NM_134420.4	Forward	(2455) tgaaagagaagtgcggtgtaga	385
		Reverse	(2839) ttcttcaggctcttaatgcaca	
Slc26a9	NM_177243.4	Forward	(1457) cactgacccctactacctctgg	425
		Reverse	(1881) tggttttcatgaagagggactt	
α-ENaC	NM_011324.2	Forward	(1496) caggcgaattattctcagttcc	451
		Reverse	(1946) ccttgggcttagggtagaagat	
β-ENaC	NM_001272023.1	Forward	(1004) acatcggtcaggaggactatgt	282
		Reverse	(1285) ggtcttggaaacaggaatgaag	
γ-ENaC	NM_011326.3	Forward	(709) gaagaaactggtgggatttcag	367
		Reverse	(1075) gaaggggttgtactcatcttcg	
Slc4a2 (Ae2)	NM_001253892.1	Forward	(3298) aacccaagattcaggaagtcaa	471
		Reverse	(3768) tctcgttgtactcatccacacc	
Slc4a3 (Ae3)	NM_001357149.1	Forward	(1612) atgaccctgatgctaaggagaa	393
		Reverse	(2004) gaatcacaatgctaccatccaa	
Slc4a4 (NBCe1)	NM_001136260.1	Forward	(2206) aaaaccagtcgctattttccaa	412
		Reverse	(2617) gggcaatggagataacagtagc	
Slc4a5 (NBCe2)	NM_001166067.1	Forward	(1814) agcctcttatcatcctcagcag	315
		Reverse	(2128) tgtaggtggtgatgaagtcagg	
Slc4a7 (NBCn1)	NM_001033270.2	Forward	(676) accctatgtggcaactctgtct	393
		Reverse	(1068) ttttctctgcttcctccacttc	
Slc4a8 (NDCBE)	NM_001347102.1	Forward	(381) gtttgaagaggatgtggaggac	434
		Reverse	(814) gatccaccttgcttagatccac	
Slc4a10 (NBCn2)	NM_001242378.1	Forward	(2967) gtgcttcgtctctcaaaggaat	445
		Reverse	(3412) cacatggcagtctttgacattt	
Slc9a1 (Nhe1)	NM_134647.4	Forward	(951) gtgcctgatagcaggagagc	202
		Reverse	(1153) ccttgtccttggacagtgct	
Slc9a2 (Nhe2)	NM_001033289.2	Forward	(2088) gcacagtcttcgggaaagtc	168
		Reverse	(2256) gtccgagtcgctgctatttc	
Slc9a3 (Nhe3)	NM_001081060.2	Forward	(2046) acagaagcggaggaatagca	199
		Reverse	(2245) tatcaattcctgccccagag	
Slc9a4 (Nhe4)	NM_177084.3	Forward	(2084) gaggaacctgccaaaatcaa	162
		Reverse	(2246) ccacgtcttcaggagaaagc	
Slc9a5 (Nhe5)	NM_001323971.2	Forward	(970) ggacaggtgggaacagtttg	182
		Reverse	(1152) ggcatagagggcagagtgag	

### Materials

BCECF-AM was obtained from Invitrogen (Carlsbad, USA); 4,4′-diisothiocyanatostilbene-2,2′-disulfonic acid disodium salt hydrate (H_2_DIDS) was from Molecular Probes (Eugene, USA); forskolin, amiloride, CFTR_inh_-172 and other standard laboratory chemicals were from Sigma (St. Louis, USA).

### Statistics

Data are presented as the means ± SD unless otherwise indicated. Tests for statistically significant differences were made with Student’s *t*-test.

## Results

Isolated bronchioles from CF mice (ΔF/ΔF mice) were used in experiments shown in Fig. 8. Isolated bronchioles from wild-type mice were used in the other experiments (Figs. 1, 2, 3, 4, 5, 6, 7).

### Basal pH_i_ in bronchiole epithelial cells and the response to luminal NH_4_^+^

When isolated bronchioles were bilaterally (bath and lumen) perfused with the standard HCO_3_^−^-buffered solution containing 25 mM HCO_3_^−^ and 5% CO_2_ (pH 7.4), basal pH_i_ was 6.94 ± 0.03 (*n* = 64, mean ± SD). When isolated bronchioles were bilaterally perfused with the standard Hepes-buffered (HCO_3_^−^–CO_2_-free) solution (pH 7.4), basal pH_i_ was 6.77 ± 0.03 (*n* = 40). Basal pH_i_ in the presence of HCO_3_^−^–CO_2_ was significantly (*p* < 0.01) higher compared to that in the absence of HCO_3_^−^–CO_2_. When NH_4_Cl (20 mM) was applied to the lumen in the presence of HCO_3_^−^–CO_2_, pH_i_ showed typical time-course changes by NH_4_^+^ pulse [40]. Addition of NH_4_Cl caused quick alkalinization (NH_3_ influx) followed by slower decline and removal of NH_4_Cl caused quick acidification (NH_3_ efflux) followed by slower recovery to the baseline (Fig. 1d). This suggests that H^+^/HCO_3_^−^ transport is active in this preparation.

### Effects of luminal HCO_3_^−^–CO_2_ removal on pH_i_ in microperfused bronchioles

**Fig. 2:**
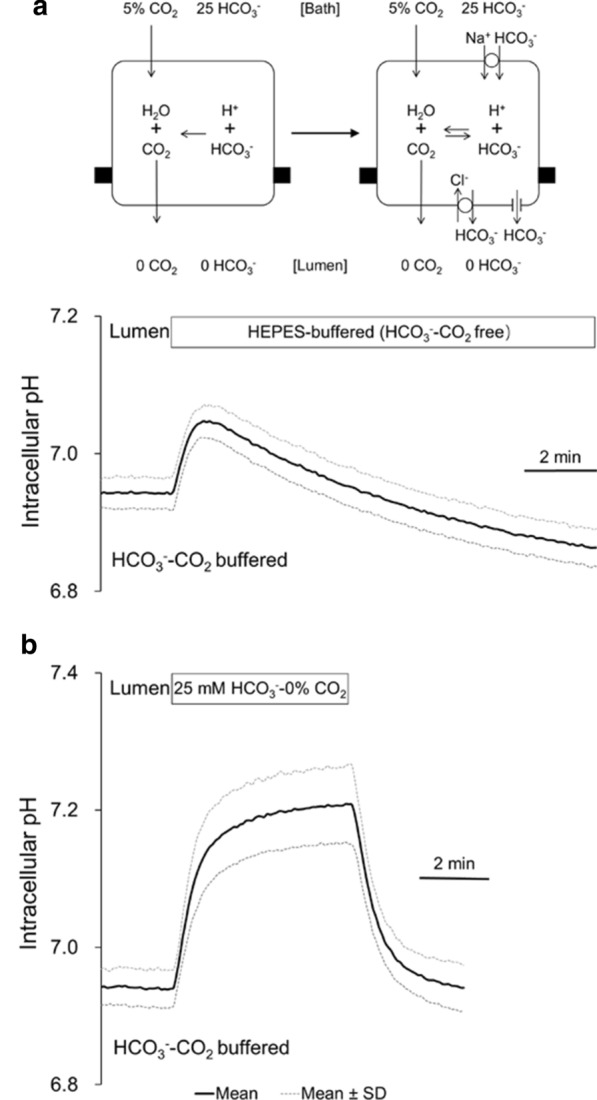
Effects of luminal HCO_3_^−^–CO_2_ removal on pH_i_ in bronchiole epithelial cells. **a** The experimental protocol used for the measurement of HCO_3_^−^ efflux across the apical membrane of microperfused bronchioles. Isolated bronchioles were first bilaterally perfused with the standard HCO_3_^−^-buffered solution and the luminal perfusate was switched to the standard Hepes-buffered HCO_3_^−^–CO_2_-free solution. It was assumed that the transient alkalinization was due to CO_2_ diffusion out of the cell and the subsequent recovery was due to HCO_3_^−^ efflux mostly across the apical membrane. Means ± SD of 8 experiments. **b** Isolated bronchioles were first bilaterally perfused with the standard HCO_3_^−^-buffered solution and the luminal perfusate was switched to the solution which first contained 25 mM HCO_3_^−^ but which was equilibrated with 100% O_2_ (pH: ~ 7.8) and thus was nominally free of CO_2_. Means ± SD of 8 experiments

When isolated bronchioles were first bilaterally perfused with the standard HCO_3_^−^-buffered solution and the luminal perfusate was switched to the standard Hepes-buffered (HCO_3_^−^–CO_2_-free) solution (Fig. 2a), pH_i_ quickly increased from 6.94 ± 0.02 to 7.05 ± 0.02 (*n* = 8) and then gradually decreased towards a value (6.86 ± 0.03) lower than the baseline in 10 min. To distinguish between the separate effects of removal of CO_2_ and HCO_3_^−^ from the lumen, a solution was prepared which first contained 25 mM HCO_3_^−^ but which was equilibrated with 100% O_2_ (pH: ~ 7.8) and thus was nominally free of CO_2_. When the luminal perfusate was switched to the 25 mM HCO_3_^−^–0% CO_2_ solution, pH_i_ quickly increased to 7.21 ± 0.06 (*n* = 8) and the alkalinization was sustained (Fig. 2b). Thus, the transient alkalinization and the subsequent recovery (acidification) by removal of luminal HCO_3_^−^–CO_2_ was most likely due to CO_2_ diffusion of out of the cell followed by HCO_3_^−^ efflux (Fig. 2a). Most of the HCO_3_^−^ efflux was probably via the apical membrane due to the steep HCO_3_^−^ gradient between the cell and the lumen (HCO_3_^−^ concentration was close to zero). H^+^ influx was not likely involved in the subsequent acidification because Na^+^–H^+^ exchanger would work for H^+^ extrusion in this condition.

### Effects of luminal application of forskolin, CFTR_inh_-172, H_2_DIDS, and amiloride on apical HCO_3_^−^ efflux in bronchiole epithelial cells

**Fig. 3:**
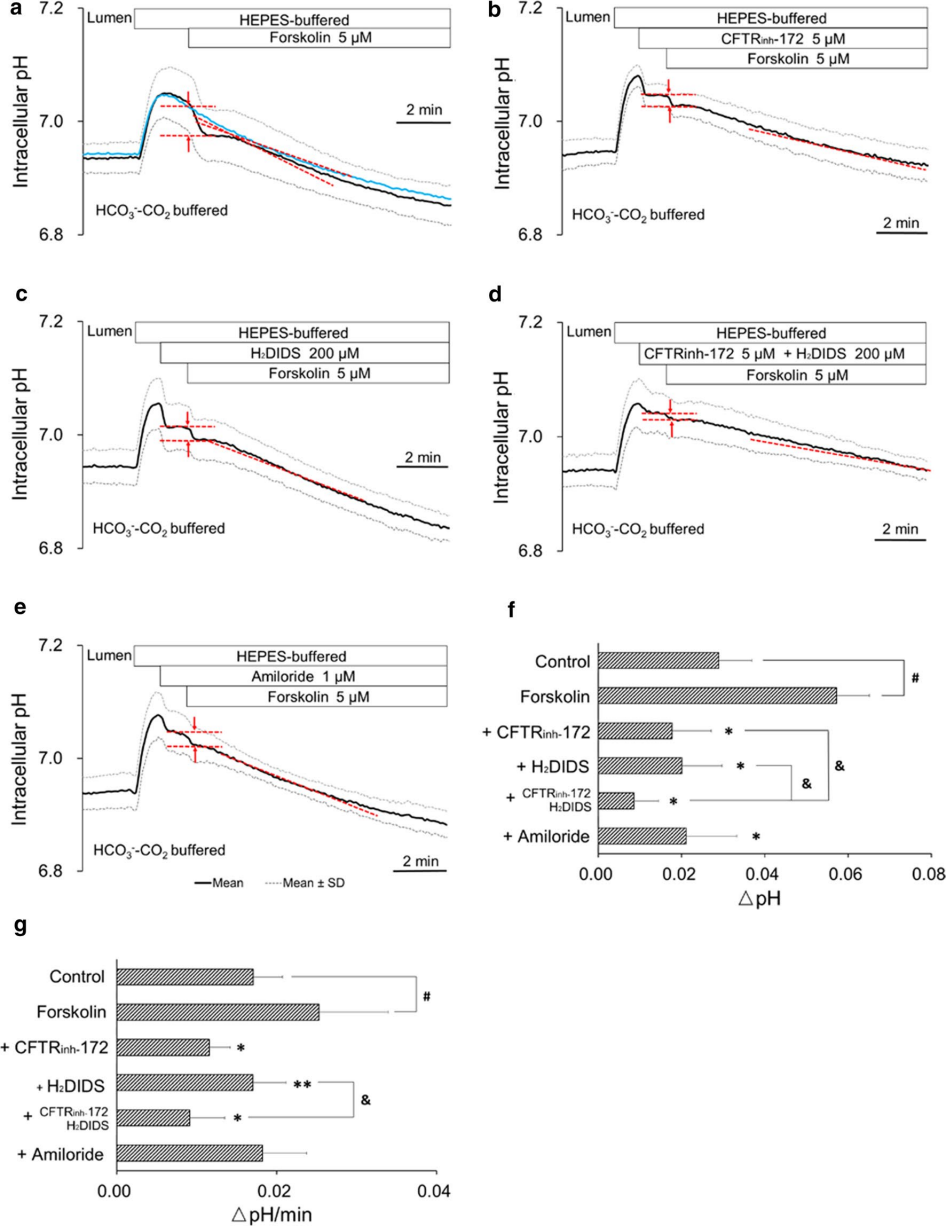
Effects of luminal application of forskolin, CFTR_inh_-172, H_2_DIDS, and amiloride on apical HCO_3_^−^ efflux. **a**–**e** Isolated bronchioles were first bilaterally perfused with the standard HCO_3_^−^-buffered solution and the luminal perfusate was switched to the standard Hepes-buffered HCO_3_^−^–CO_2_-free solution. After HCO_3_^−^–CO_2_ was removed from the luminal perfusate, forskolin (5 μM) was applied to the lumen. Time course changes of pH_i_ in the absence (**a**) or presence of CFTR_inh_-172 (5 μM) in the lumen (**b**), H_2_DIDS (200 μM) in the lumen (**c**), both CFTR_inh_-172 (5 μM) and H_2_DIDS (200 μM) in the lumen (**d**), or amiloride (1 μM) in the lumen (**e**) are shown as means ± SD of 8 experiments, respectively. The blue line in **a** indicates mean change of pH_i_ without forskolin stimulation as a reference. **f** Early-phase pH_i_ decline (ΔpH for 1 min) just after forskolin stimulation. ^#^*p* < 0.01 compared with control (without forskolin stimulation). *p < 0.01 compared with forskolin alone. ^&^*p* < 0.05. **g** Late-phase pH_i_ decline (ΔpH/min at midpoint pH_i_ of 6.95) under forskolin stimulation (red dashed lines in a-e). ^#^*p* < 0.05 compared with control (without forskolin stimulation). *p < 0.01, **p < 0.05 compared with forskolin alone. ^&^*p* < 0.01

The mechanisms for HCO_3_^−^ efflux across the apical membrane were examined using the protocol of Fig. 2a. After HCO_3_^−^–CO_2_ was removed from the luminal perfusate, forskolin (5 μM), the activator of adenylate cyclase, was applied to the lumen as indicated (Fig. 3a). Stimulation with forskolin transiently accelerated the pH_i_ decline by 97% (*p* < 0.01) (*n* = 8, Fig. 3a and f) of control (without forskolin stimulation: blue line in Fig. 3a). The late phase of pH_i_ decline (at midpoint pH_i_ of 6.95) was also accelerated by 47% (*p* < 0.05) (Fig. 3a and g) of control. The data suggest that elevation of intracellular cAMP activates HCO_3_^−^ secretion in a biphasic manner: initial large response followed by sustained activation, in mice bronchiole epithelial cells.

CFTR_inh_-172 (5 μM) and H_2_DIDS (200 μM) in the lumen inhibited the forskolin-stimulated transient pH_i_ decline by 69% (*n* = 8, *p* < 0.01) (Fig. 3b and f) and 65% (*n* = 8, *p* < 0.01) (Fig. 3c and f), respectively. Luminal CFTR_inh_-172 and H_2_DIDS also slowed down the late phase of pH_i_ decline (at midpoint pH_i_ of 6.95) by 54% (*p* < 0.01) (Fig. 3b and g) and 33% (*p* < 0.05) (Fig. 3c and g), respectively. The data suggest that both CFTR and H_2_DIDS-sensitive HCO_3_^−^ transporter and/or HCO_3_^−^-permeable anion channel partly mediate cAMP-stimulated HCO_3_^−^ secretion. The forskolin-stimulated transient pH_i_ decline in the presence of both CFTR_inh_-172 and H_2_DIDS (Fig. 3d and f) was significantly (*p* < 0.05) smaller compared to that in the presence of CFTR_inh_-172 or H_2_DIDS alone (Fig. 3b, c, and f). The late-phase of pH_i_ decline in the presence of both CFTR_inh_-172 and H_2_DIDS (Fig. 3d and g) was significantly (*p* < 0.05) slower compared to that in the presence of H_2_DIDS alone (Fig. 3c and g).

Luminal application of CFTR_inh_-172 and H_2_DIDS by themselves induced a transient small dip of pH_i_ (Fig. 3b and c). The transient pH_i_ dip largely disappeared when CFTR_inh_-172 and H_2_DIDS were simultaneously applied to the lumen (Fig. 3d). This suggests that CFTR and H_2_DIDS-sensitive HCO_3_^−^ transporter/channel compensate each other for apical HCO_3_^−^ efflux. We speculate on the mechanisms as follows. CFTR inhibition would hyperpolarize the cell, which would induce transient HCO_3_^−^ efflux via a HCO_3_^−^-permeable anion channel or an electrogenic HCO_3_^−^ transporter (such as 1Cl^−^–2HCO_3_^−^ exchanger). If H_2_DIDS-sensitive HCO_3_- transport is electrogenic, luminal H_2_DIDS would hyperpolarize the cell, which would induce transient HCO_3_^−^ efflux via CFTR.

To examine the role of ENaC in HCO_3_^−^ secretion, a relatively low concentration of amiloride (1 μM) [32] was applied to the lumen. Amiloride (1 μM) in the lumen inhibited the forskolin-stimulated transient pH_i_ decline by 63% (*n* = 8, *p* < 0.01) (Fig. 3e and f), but did not significantly affect the late phase of pH_i_ decline (Fig. 3e and g). The data suggest that ENaC is involved in the regulation of HCO_3_^−^ secretion. The transient dip of pH_i_ by luminal amiloride (Fig. 3e) likely indicates apical HCO_3_^−^ efflux which is accelerated by membrane hyperpolarization.

When isolated bronchioles were bilaterally perfused with the standard HCO_3_^−^-buffered solution, application of CFTR_inh_-172 (5 μM) to the lumen caused a transient increase of pH_i_ by 0.022 ± 0.003 unit (*n* = 8, data not shown). The pH_i_ increase was not observed in the absence of HCO_3_^−^–CO_2_ and enhanced by 77% (*p* < 0.05) by stimulation with forskolin (5 μM) (data not shown). The data suggest that CFTR is involved in HCO_3_^−^ secretion in a physiological condition.

### Effects of luminal Cl^−^ removal on pH_i_ in bronchiole epithelial cells

**Fig. 4:**
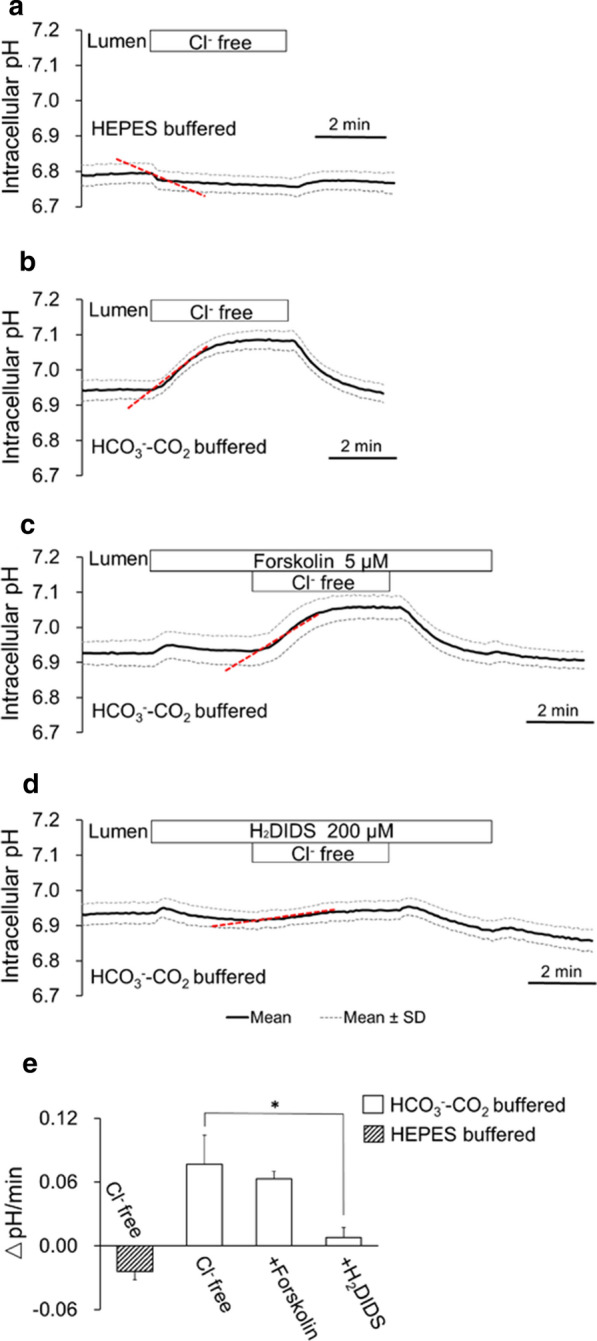
Effects of luminal Cl^−^ removal on pH_i_ in bronchiole epithelial cells. **a**–**d** Isolated bronchioles were first bilaterally perfused with the standard Hepes-buffered HCO_3_^−^–CO_2_-free solution (**a**) or the standard HCO_3_^−^-buffered solution (**b**–**d**). Luminal Cl^−^ was removed by replacement with gluconate in the absence (**a**, **b**) or presence of luminal forskolin (5 μM) (**c**) or luminal H_2_DIDS (200 μM) (**d**). Time course changes of pH_i_ are shown as means ± SD of 8 experiments, respectively. **e** The activity of apical Cl^−^–HCO_3_^−^ exchange is shown as the initial rate of pH_i_ increase upon luminal Cl^−^ removal (red dashed lines in **a**–**d**). *p < 0.01

To examine the activity of Cl^−^–HCO_3_^−^ exchange in the apical membrane, effects of luminal Cl^−^ removal on pH_i_ were examined. When isolated bronchioles were bilaterally perfused with the standard Hepes-buffered HCO_3_^−^–CO_2_-free solution, removal of luminal Cl^−^ (by replacement with gluconate) caused a slight decline of pH_i_ (Fig. 4a and e). In contrast, when isolated bronchioles were bilaterally perfused with the standard HCO_3_^−^-buffered solution, luminal Cl^−^ removal caused a reversible increase of pH_i_ by 0.14 ± 0.03 unit (*n* = 8) over ~ 4 min period (Fig. 4b and e), most likely due to influx of luminal HCO_3_^−^ in exchange for intracellular Cl^−^. When the activity of apical Cl^−^–HCO_3_^−^ exchange is shown as the initial rate of pH_i_ increase upon luminal Cl^−^ removal, the activity is not affected by forskolin (5 μM) in the lumen (Fig. 4c and e) and largely (*p* < 0.01) inhibited by H_2_DIDS (200 μM) in the lumen (Fig. 4d and e). The data suggest that H_2_DIDS-sensitive Cl^−^–HCO_3_^−^ exchanger is localized in the apical membrane.

### Na^+^-dependent H^+^ extrusion across the apical membrane of bronchiole epithelial cells

**Fig. 5:**
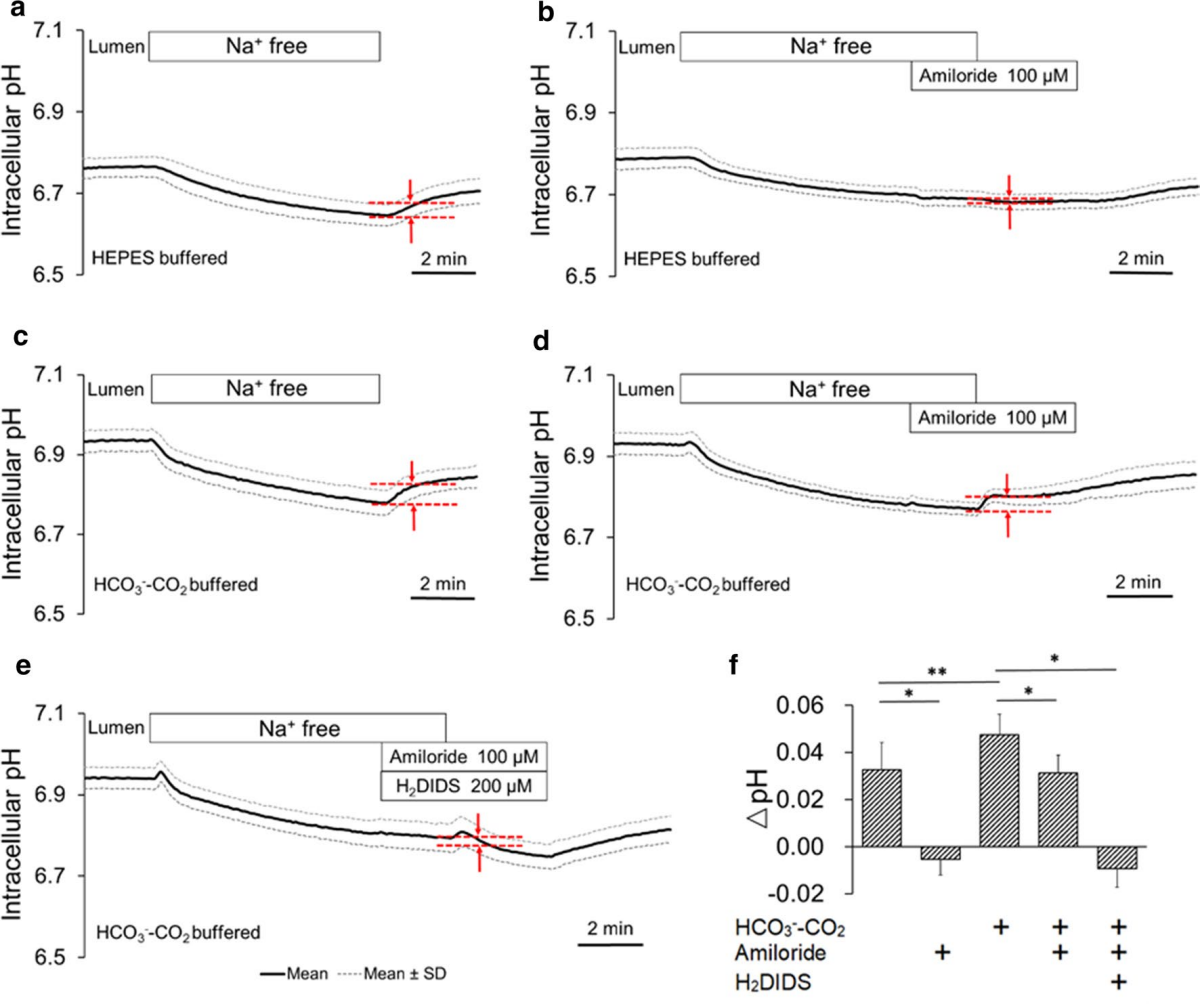
Na^+^-dependent H^+^ extrusion across the apical membrane of bronchiole epithelial cells. **a**–**e** Isolated bronchioles were first bilaterally perfused with the standard Hepes-buffered HCO_3_^−^–CO_2_-free solution (**a**, **b**) or the standard HCO_3_^−^-buffered solution (**c**–**e**). Luminal Na^+^ was removed by replacement with NMDG and restored to the lumen in the absence (**a**, **c**) or presence of luminal amiloride (100 μM) (**b**, **d**) or combination of luminal amiloride (100 μM) and H_2_DIDS (200 μM) (**e**). Time course changes of pH_i_ are shown as means ± SD of 8 experiments. **f** The activity of luminal Na^+^-dependent H^+^ extrusion is shown as the initial increase/decrease of pH_i_ (ΔpH for 1 min) upon restoration of luminal Na^+^. *p < 0.01, **p < 0.05

To examine whether Na^+^–H^+^ exchanger (NHE) and Na^+^–HCO_3_^−^ cotransporter (NBC) are localized in the apical membrane, luminal Na^+^-dependent H^+^ extrusion was examined. In the absence of HCO_3_^−^–CO_2_, removal of luminal Na^+^ (by replacement with NMDG) caused a continuous decline of pH_i_ and restoration of Na^+^ to the lumen caused a recovery (Fig. 5a). When the activity of luminal Na^+^-dependent H^+^ extrusion is shown as the initial pH_i_ increase (ΔpH for 1 min) upon restoration of luminal Na^+^, the activity was completely (*p* < 0.01) inhibited by amiloride (100 μM) in the lumen (Fig. 5b and f). The data suggest that NHE is localized in the apical membrane.

The activity of luminal Na^+^-dependent H^+^ extrusion in the presence of HCO_3_^−^–CO_2_ (Fig. 5c and f) was significantly (*p* < 0.05) greater compared to that in the absence of HCO_3_^−^–CO_2_ (Fig. 5a and f), partially inhibited by amiloride (100 μM) in the lumen (Fig. 5d and f), and completely inhibited by a combination of amiloride (100 μM) and H_2_DIDS (200 μM) in the lumen (Fig. 5e and f). The data suggest that NBC is localized in the apical membrane.

### Effects of luminal amiloride on pH_i_ in bronchiole epithelial cells

**Fig. 6:**
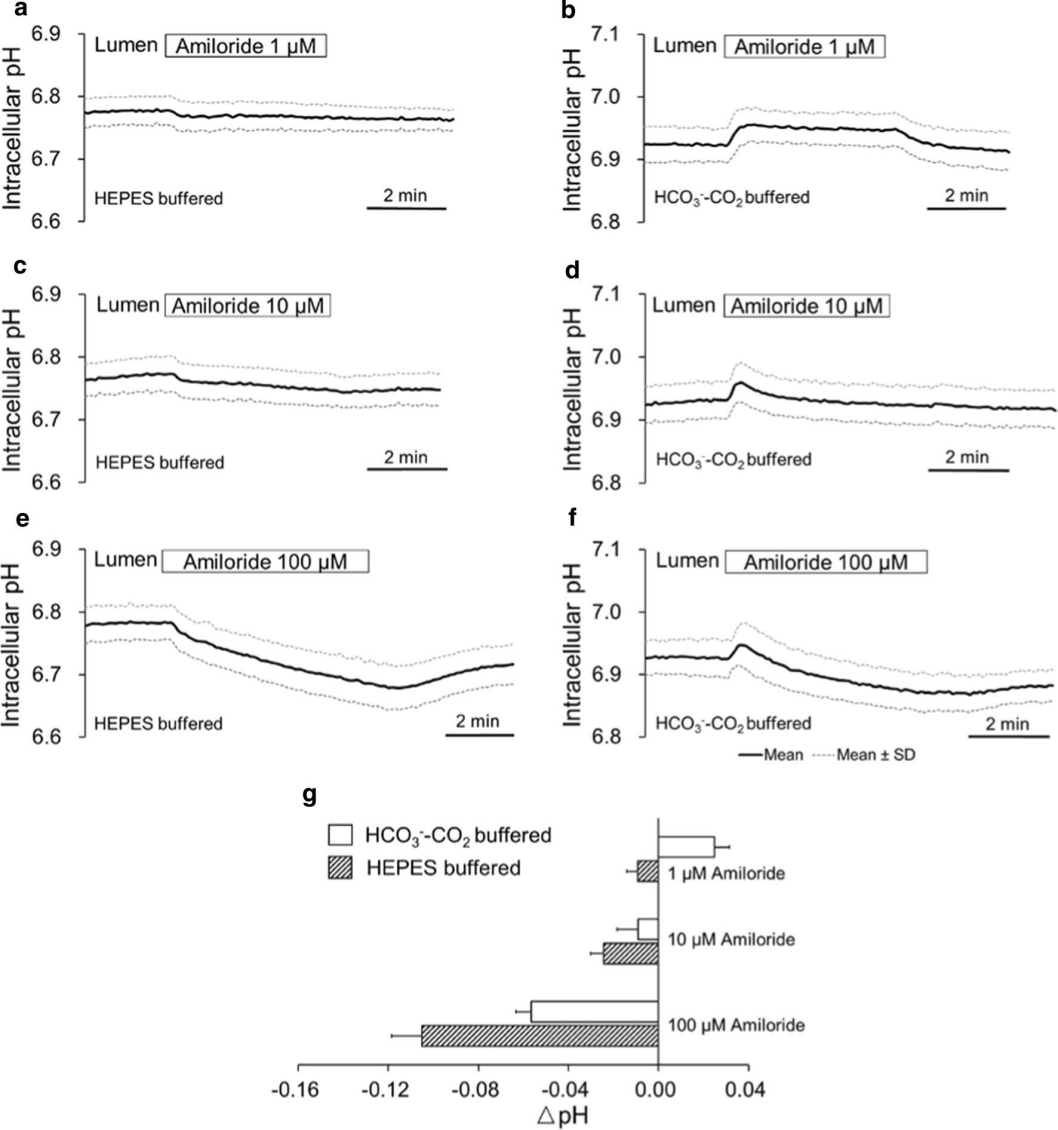
Effects of luminal amiloride on pH_i_ in bronchiole epithelial cells. **a**–**f** Isolated bronchioles were first bilaterally perfused with the standard Hepes-buffered HCO_3_^−^–CO_2_-free solution (**a**, **c**, **e**) or the standard HCO_3_^−^-buffered solution (**b**, **d**, **f**). Amiloride was applied to the lumen as indicated at concentrations of 1 μM (**a**, **b**), 10 μM (**c**, **d**) or 100 μM (**e**, **f**). Time course changes of pH_i_ are shown as means ± SD of 8 experiments. **g** Increase or decrease of pH_i_ (ΔpH for 5 min) by luminal application of amiloride at various concentrations

While lower concentrations of amiloride inhibit ENaC with IC50 of 1 μM [32], higher concentrations of amiloride (0.5–1 mM) also inhibit apical NHE in human bronchial epithelium [49]. Figure 6 shows the effects of various concentrations of luminal amiloride (1, 10, and 100 μM) on basal pH_i_ (Fig. 6). To examine the relative contribution of ENaC and apical NHE in H^+^/HCO_3_^−^ transport in a physiological condition, we examined concentration-dependent effects of luminal amiloride rather than a more specific inhibitor of NHE such as ethylisopropyl amiloride (EIPA).

In the absence of HCO_3_^−^–CO_2_, luminal application of amiloride caused concentration-dependent decline of pH_i_ (Fig. 6a, c, e, g).

In the presence of HCO_3_^−^–CO_2_, luminal application of 1 μM amiloride caused an increase in pH_i_ by 0.03 ± 0.01 (*n* = 8, Fig. 6b and g). Luminal 100 μM amiloride caused a transient increase followed by a continuous decline in pH_i_ by 0.06 ± 0.01 (*n* = 8) in 5 min (Fig. 6f and g). Luminal 10 μM amiloride (Fig. 5d and g) caused an intermediate pattern of pH_i_ changes of those by 1 μM and 100 μM amiloride.

Thus, the effects of lower concentration of apical amiloride on basal pH_i_ were dependent on the presence of HCO_3_^−^–CO_2_, which suggests that ENaC is involved in the regulation of HCO_3_^−^ transport. The data also indicate that apical NHE is involved in the regulation of basal pH_i_.

### Messenger RNA expression of ion transporters and channels in bronchiole epithelial cells

**Fig. 7:**
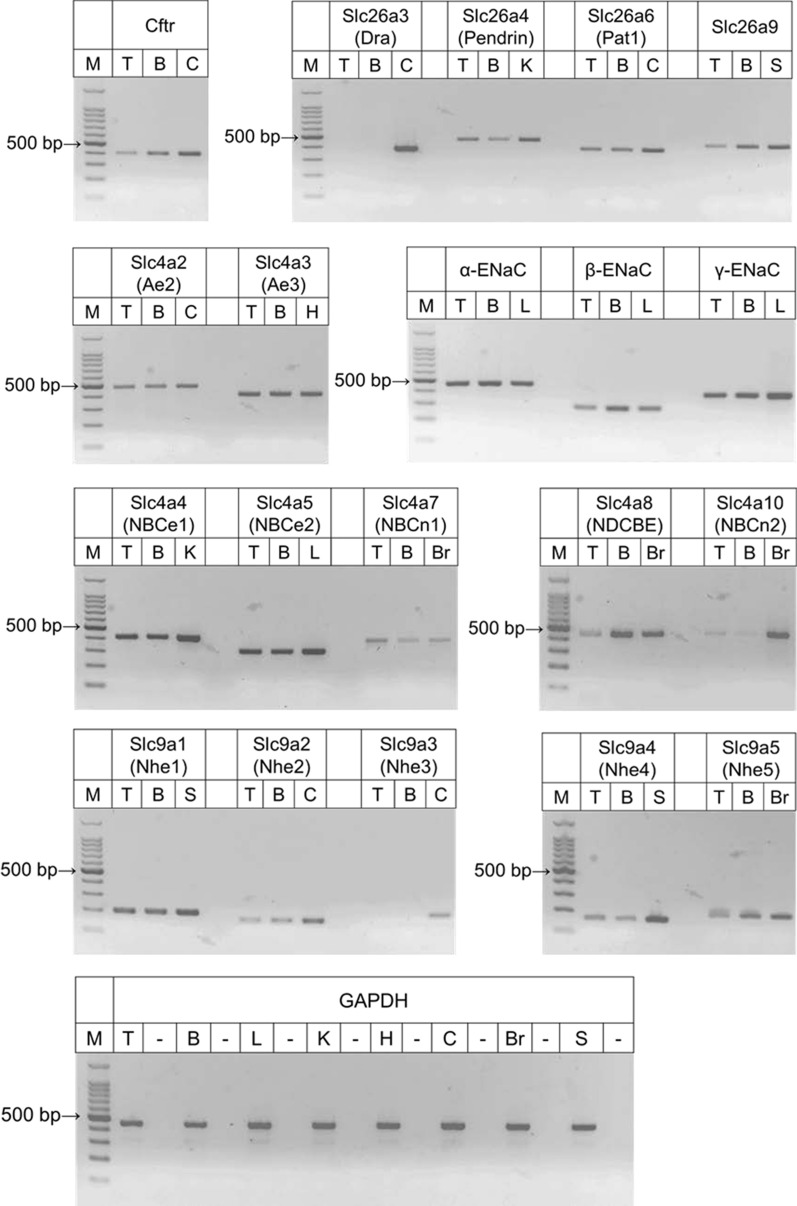
Messenger RNA expression of ion transporters and channels in bronchiole epithelial cells. Messenger RNA was extracted from tracheal mucosa (T), isolated bronchioles (B), colon (C), kidney (K), stomach (S), heart (H), lung (L), and brain (Br) and reverse transcribed. PCR was performed using each cDNA as template and with gene-specific primers (Table 1). GAPDH was used as a reference. ‒: PCR was performed in the absence of RT enzyme. M: 100-bp DNA ladder

Expression of Cftr, ENaC subunits, and Slc4, Slc9, and Slc26 families of transporters in isolated bronchioles and tracheal mucosa was examined by RT-PCR (Fig. 7). Amplified fragments from Cftr, α, β, γ subunits of ENaC, Slc4a2 (Ae2), Slc4a3 (Ae3), Slc4a4 (NBCe1), Slc4a5 (NBCe2), Slc4a7 (NBCn1), Slc4a8 (NDCBE), Slc4a10 (NBCn2), Slc9a1 (Nhe1), Slc9a2 (Nhe2), Slc9a4 (Nhe4), Slc9a5 (Nhe5), Slc26a4 (Pendrin), Slc26a6 (Pat1), and Slc26a9 were detected in isolated bronchioles and tracheal mucosa. Fragments from Slc9a3 (Nhe3) and Slc26a3 (Dra) were not detected in isolated bronchioles and tracheal mucosa.

### Basal pH_i_ and apical HCO_3_^−^ efflux in bronchiole epithelial cells from CF mice

**Fig. 8:**
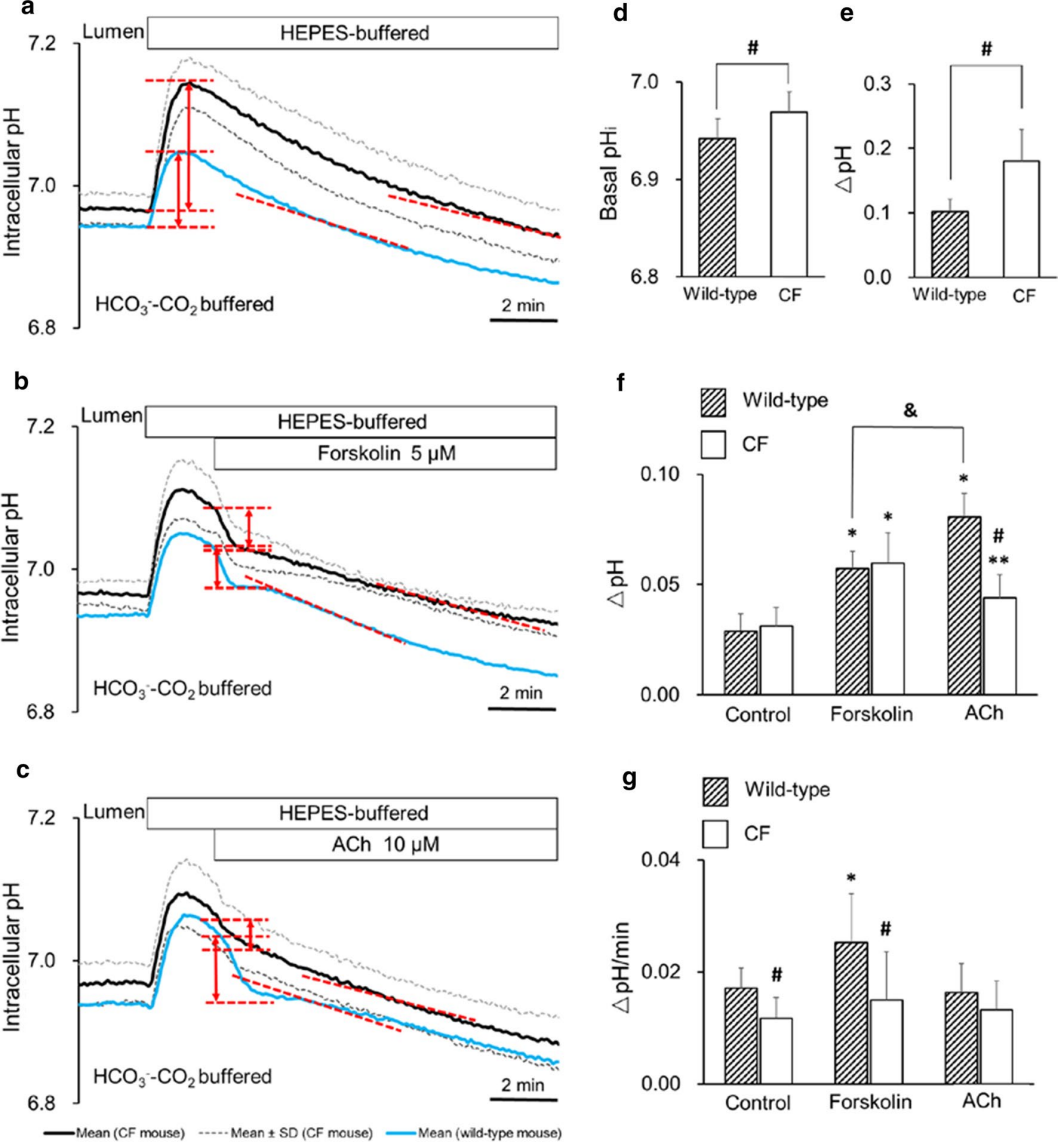
Apical HCO_3_^−^ efflux in bronchiole epithelial cells from CF mice. **a**–**c** Isolated bronchioles from ΔF/ΔF mice were first bilaterally perfused with the standard HCO_3_^−^-buffered solution and the luminal perfusate was switched to the standard Hepes-buffered HCO_3_^−^–CO_2_-free solution. After HCO_3_^−^–CO_2_ was removed from the luminal perfusate, forskolin (5 μM, **b**) or ACh (10 μM, **c**) was applied to the lumen. Means ± SD of 5–6 experiments, respectively. Blue lines indicate mean changes of pH_i_ in wild-type bronchioles as references. **d** Basal pH_i_ in the presence of HCO_3_^−^–CO_2_ in wild-type (*n* = 8) and ΔF/ΔF (*n* = 6) bronchioles. ^#^*p* < 0.05. **e** Transient increase of pH_i_ (ΔpH) by removal of luminal HCO_3_^−^–CO_2_ in wild-type and ΔF/ΔF bronchioles. ^#^*p* < 0.01. **f** Early-phase pH_i_ decline (ΔpH for 1 min) just after stimulation with forskolin or acetylcholine. ^#^*p* < 0.01 compared to wild-type. *p < 0.01, **p < 0.05 compared with control (without stimulation). ^&^*p* < 0.01. **g** Late-phase pH_i_ decline (ΔpH/min at midpoint pH_i_ of 6.95) under stimulation with forskolin or ACh (red dashed lines in **a**–**c**). *p < 0.05 compared with control (without stimulation). ^#^*p* < 0.05 compared to wild-type

Basal pH_i_ in the presence of HCO_3_^−^–CO_2_ in isolated bronchioles from ΔF/ΔF mice (6.97 ± 0.02, *n* = 6) was slightly but significantly (*p* < 0.05) higher compared to bronchioles from wild-type mice (6.94 ± 0.02, *n* = 8, blue line) (Fig. 8a and d). Initial increase of pH_i_ (ΔpH) by removal of luminal HCO_3_^−^–CO_2_ was also significantly (*p* < 0.01) greater in ΔF/ΔF bronchioles compared to wild-type bronchioles (Fig. 8a and e). The data suggest that basal HCO_3_^−^ secretion is impaired in CF bronchioles. The rate of pH_i_ decline at midpoint pH_i_ of 6.95 was significantly (*p* < 0.05) slower in CF bronchioles compared to wild-type bronchioles (Fig. 8a and g).

Stimulation with luminal forskolin (5 μM) transiently accelerated pH_i_ decline (apical HCO_3_^−^ efflux) in ΔF/ΔF bronchioles (Fig. 8b and f) and the acceleration was comparable to wild-type bronchioles (blue line). Forskolin failed to accelerate the late phase of pH_i_ decline (at midpoint pH_i_ of 6.95) in ΔF/ΔF bronchioles (Fig. 8b and g). The data suggest that cAMP stimulation transiently activated HCO_3_^−^ secretion in CF bronchioles probably via activation of a HCO_3_^−^-permeable anion channel or a HCO_3_^−^ transporter, but failed to induce sustained increase of HCO_3_^−^ secretion.

Luminal application of ACh induced a transient increase of transepithelial ion current in mice and pig tracheal epithelium [16, 21]. Application of ACh (10 μM) to the lumen transiently accelerated pH_i_ decline (apical HCO_3_^−^ efflux) in wild-type bronchioles (blue line in Fig. 8c) and the acceleration was greater than forskolin (Fig. 8f, *p* < 0.01). The ACh-induced transient acceleration of pH_i_ decline was reduced by 45% (*p* < 0.01) in ΔF/ΔF bronchioles (Fig. 8c and f). Luminal ACh did not affect the late phase of pH_i_ decline in both wild-type and ΔF/ΔF bronchioles (Fig. 8c and g). The data indicate that ACh stimulation transiently activated HCO_3_^−^ secretion in wild-type bronchioles and that the ACh-induced enhancement of HCO_3_^−^ secretion was substantially reduced in CF bronchioles. This suggests that CFTR partly mediates ACh-induced HCO_3_^−^ secretion in addition to CaCC in mice bronchiole epithelial cells.

## Discussion

In the present study, HCO_3_^−^ transport in surface epithelial cells of native bronchioles was studied by measuring pH_i_ in luminally microperfused freshly dissected mice bronchioles. HCO_3_^−^ transport in bronchioles from CF mice was also studied. Although some connective tissue was attached to the outside of bronchioles (Fig. 1), surface epithelial cells were successfully loaded with BCECF from the lumen and pH_i_ was measured as long as 30 min. The present study focused on HCO_3_^−^/H^+^ transport across the apical membrane, since rapid exchange of luminal solutions was achieved in our preparation [22].

### Intracellular pH in surface epithelial cells of mice bronchioles

Human and rodent bronchioles are lined with columnar to cuboidal epithelium which is composed of ciliated and nonciliated (Clara) cells [35]. In the present study, basal pH_i_ of surface epithelial cells in isolated mice bronchioles was ~ 6.94 in bilateral (bath and lumen) presence of 25 mM HCO_3_^−^ and 5% CO_2_. The value is similar to the basal pH_i_ of cultured human nasal epithelial cells (~ 6.94) in the same experimental condition [37]. The relatively low basal pH_i_ likely resulted from higher pCO_2_ in the lumen compared to the physiological in vivo situation where the luminal side of the epithelial layer is exposed to air.

### Ion transporters and channels localized in the apical membrane of bronchiole epithelial cells

**Fig. 9:**
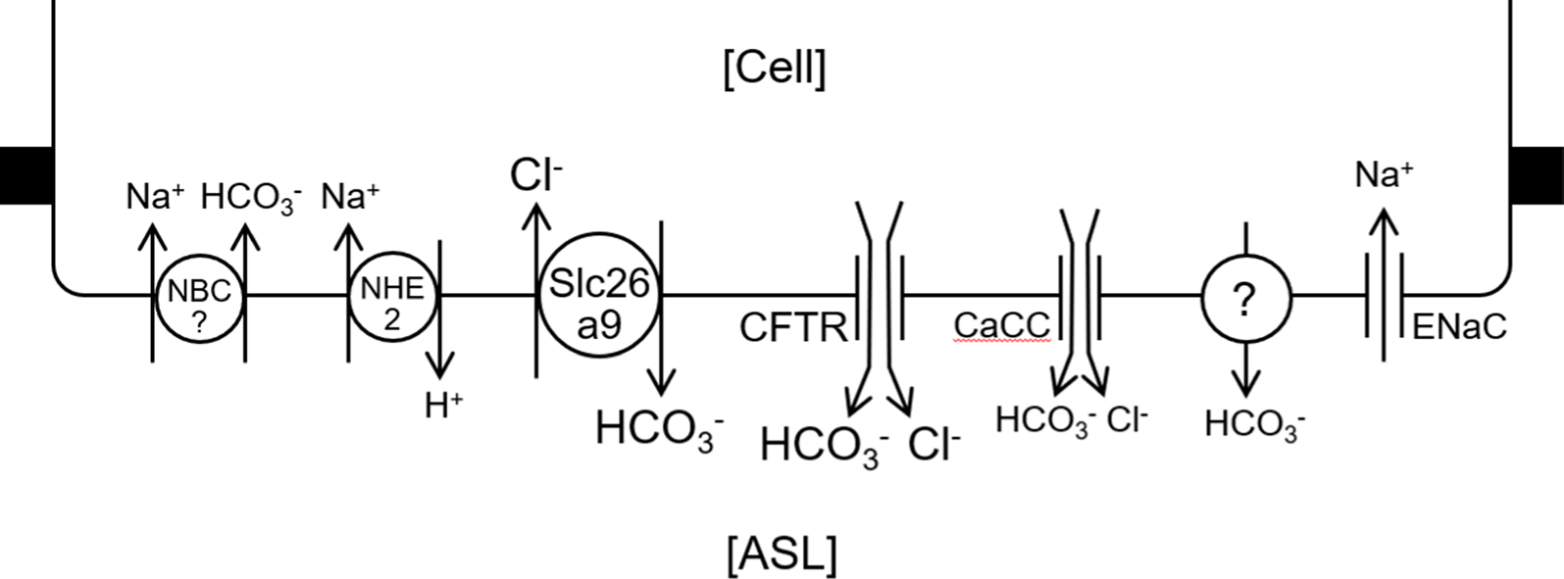
A hypothetical model for H^+^/HCO_3_^−^ transport across the apical membrane of airway surface epithelial cells in mice bronchiole. HCO_3_^−^ secretion across the apical membrane is largely mediated by CFTR and Slc26a9 Cl^−^–HCO_3_^−^ exchanger. CaCC is also involved in HCO_3_^−^ secretion. An unknown HCO_3_^−^-permeable anion channel or HCO_3_^−^ transporter is upregulated in CF bronchioles. ENaC is involved in the regulation of HCO_3_^−^ transport but the mechanisms are not clear. NHE2 and NBC contribute to the regulation of intracellular and ASL pH

In the present study, functional studies suggested that CFTR and H_2_DIDS-sensitive HCO_3_^−^ transporter and/or HCO_3_^−^-permeable anion channel mediate cAMP-stimulated HCO_3_^−^ secretion and ENaC, H_2_DIDS-sensitive Cl^−^–HCO_3_^−^ exchangers, NHE, and NBC are involved in HCO_3_^−^/H^+^ transport across the apical membrane of surface epithelial cells of mice bronchioles (Fig. 9). This is supported by mRNA expression of Cftr, ENaC subunits, and Slc4, Slc9, and Slc26 families of transporters (Fig. 7).

The activity of H_2_DIDS-sensitive Cl^−^–HCO_3_^−^ exchanger was detected in the apical membrane (Fig. 4) and probably mediated part of cAMP-stimulated HCO_3_^−^ secretion (Fig. 3c). The candidate molecules are Slc4a2 (Ae2), Slc4a3 (Ae3), Slc26a4 (Pendrin), Slc26a6, and Slc26a9 of which mRNA expression was detected in isolated bronchioles (Fig. 7). In human bronchial epithelia, SLC26A4 (Pendrin) colocalized with CFTR in the apical membrane of ciliated surface cells and mediated most of HCO_3_^−^ secretion when pretreated with IL-4 [24]. SLC26A9 is prominently expressed in brain and on apical membrane of airway epithelial cells and gastric mucosa [3, 31]. A missense variant of SLC26A9 found in a patient of diffuse bronchiectasis failed to activate CFTR in a heterologous expression system [7]. While Slc26a4 (Pendrin) is H_2_DIDS-insensitive, Slc26a9 is sensitive to H_2_DIDS. Thus, Slc26a9 is likely the major apical Cl^−^–HCO_3_^−^ exchanger in mice bronchioles.

Apical H^+^ secretion via H^+^/K^+^ ATPase and vacuolar H^+^-ATPase was reported in airways [52]. Our present study identified the activities of NHE and NBC in the apical membrane of mice bronchioles (Figs. 5, 6) which may contribute to the regulation of intracellular and ASL pH.

NHE activity was detected in the apical membrane and mediated H^+^ secretion in tracheal epithelial cells from sheep [1]. The candidate molecules of apical NHE in mice bronchioles are Slc9a1 (Nhe1), Slc9a2 (Nhe2), Slc9a4 (Nhe4), and Slc9a5 (Nhe5) of which mRNA expression was detected (Fig. 7). NHE2 is known to be expressed in the lung, predominantly localized to the apical membrane of epithelial cells [19], and relatively sensitive to amiloride [51]. Thus, NHE2 is likely the major apical Na^+^–H^+^ exchanger in mice bronchioles.

While SlC4A4 (NBCe1) and SLC4A5 (NBCe2) were identified in the basolateral membrane of Calu-3 cells [27], NBC isoforms have not been identified in the apical membrane of airway epithelium. Messenger RNA of all NBC isoforms: Slc4a4 (NBCe1), Slc4a5 (NBCe2), Slc4a7 (NBCn1), Slc4a8 (NDCBE), and Slc4a10 (NBCn2) was detected in isolated mice bronchioles (Fig. 7). Our present study cannot identify the membrane localization of the NBC isoforms.

### Mechanisms and regulation of HCO_3_^−^ secretion in bronchiole epithelial cells

Surface airway epithelial cells as well as serous cells of the submucosal glands secrete Cl^−^ and HCO_3_^−^ in response to agents increasing intracellular cAMP (VIP, noradrenaline, etc.) and/or Ca^2+^ (ACh, histamine, etc.) [41]. It is generally accepted that cAMP-mediated secretion involves CFTR and Ca^2+^-mediated secretion involves CaCC encoded by TMEM16A/ANO1 [15]. Cyclic AMP- and Ca^2+^-mediated agonists independently and additively increased HCO_3_^−^ secretion in human bronchioles [45]. However, it has been noted that muscarinic responses of fluid secretion are reduced in submucosal glands from patients with cystic fibrosis [42] and a recent study demonstrated a crosstalk of CFTR and TMEM16A in CFBE cells [29].

We assume that continuous decline of pH_i_ following alkaline load (Figs. 2, 3, 8) demonstrates time course of HCO_3_^−^ secretion into the lumen which is perfused with the HCO_3_^−^-free solution. Forskolin biphasically stimulated HCO_3_^−^ secretion: transiently accelerated HCO_3_^−^ secretion just after application and increased the rate of steady-state HCO_3_^−^ secretion (Fig. 3a). ACh transiently accelerated HCO_3_^−^ secretion, but did not increase the steady-state HCO_3_^−^ secretion (Fig. 8c). The data indicate that both cAMP-mediated and Ca^2+^-mediated pathways are involved in HCO_3_^−^ secretion in mice bronchiole epithelial cells.

Luminal CFTR_inh_-172 and H_2_DIDS substantially inhibited both transient and steady-state phases of forskolin-stimulated HCO_3_^−^ secretion (Fig. 3). CFTR was localized not only in serous cells of submucosal glands [17, 23], but also in the apical membrane of surface epithelium of proximal to distal airways in human [26]. GlyH101-sensitive HCO_3_^−^ transport was detected in human bronchioles [45]. Our present data suggest that both CFTR and H_2_DIDS-sensitive HCO_3_^−^ transporter (likely SLC26A9 Cl^−^–HCO_3_^−^ exchanger shown in Fig. 4) and/or HCO_3_^−^-permeable anion channel are involved in apical HCO_3_^−^ secretion (Fig. 9).

A relatively low concentration of amiloride in the lumen inhibited transient phase of forskolin-stimulated HCO_3_^−^ secretion (Fig. 3e). The data suggest that ENaC is involved in the regulation of HCO_3_^−^ transport, which is consistent with amiloride (1 μM)-induced pH_i_ increase in the presence of HCO_3_^−^–CO_2_ (Fig. 6). The cellular mechanisms for the involvement of ENaC in HCO_3_^−^ secretion are not clear.

### HCO_3_^−^ secretion in CF bronchiole epithelial cells

ASL pH was more acidic in trachea of CF pigs under basal and methacholine-stimulated conditions [38]. Lower pH of ASL was also observed in nasal epithelium of CF patients [34, 52], while the other study did not find differences in ASL pH of bronchus between CF patients and control [43]. Combination of forskolin and 3-isobutyl-1-methylxanthine alkalinized ASL of cultured bronchial epithelium of normal subjects but acidified CF ASL [12].

In the present study, HCO_3_^−^ secretion was studied in bronchioles isolated from a CF mouse model in which the F508del mutation (most frequent pathogenic variant of CFTR) was introduced (ΔF mouse) (Fig. 8). Although CF mice do not display severe lung disease as observed in humans, an impaired ability to stretch/expand the peripheral lung compartment and increased distances between gas exchange surfaces which are early pulmonary phenotype of human CF were found in young (8–16 weeks old) ΔF/ΔF mice [14]. Our present study demonstrated higher level of basal pH_i_ in the presence of HCO_3_^−^–CO_2_ and larger increase of pH_i_ by removal of luminal HCO_3_^−^–CO_2_ in CF bronchioles (Fig. 8), which indicate that basal HCO_3_^−^ secretion is reduced in CF distal airways.

The effects of forskolin and ACh on HCO_3_^−^ secretion in CF bronchioles (Fig. 8) were unexpected. While forskolin stimulation transiently accelerated HCO_3_^−^ secretion in CF bronchioles (comparable to wild-type bronchioles, Fig. 8b), ACh-induced acceleration of HCO_3_^−^ secretion was substantially reduced in CF bronchioles (Fig. 8c). The data are consistent with the presence of a crosstalk of cAMP- and Ca^2+^-mediated pathways of HCO_3_^−^ secretion. The data also suggest that a cAMP-activated HCO_3_^−^-permeable anion channel or HCO_3_^−^ transporter was upregulated in CF bronchioles.

The present study has some limitations. (1) The intracellular buffering capacity is not measured and the rate of H^+^/HCO_3_^−^ flux is not inferred from changes in pH_i_. (2) Information of membrane potential is not available and the electrochemical potential gradient for HCO_3_^−^ across the apical membrane is not accurately predicted. (3) RT-PCR of isolated bronchioles does not identify the cell types (ciliated or nonciliated) and the membrane (apical or basolateral) in which transporters/channels are located.

In summary, we have characterized HCO_3_^−^/H^+^ transport across the apical membrane of surface epithelial cells of native mice bronchioles. We have demonstrated that cAMP-mediated and Ca^2+^-mediated pathways are involved in HCO_3_^−^ secretion and that apical HCO_3_^−^ secretion is largely mediated by CFTR and Cl^−^–HCO_3_^−^ exchange. The impairment of HCO_3_^−^ secretion in CF bronchioles may be related to the pathogenesis of early lung disease in CF.

## Data Availability

All data generated or analyzed during this study are included in the manuscript.

## References

[1] Acevedo M, Steele LW (1993). Na^+^-H^+^ exchanger in isolated epithelial tracheal cells from sheep. Involvement in tracheal proton secretion. Exp Physiol.

[2] Al-Bazzaz FJ, Tarka C, Farah M (1991). Microperfusion of sheep bronchioles. Am J Physiol.

[3] Alper SL, Sharma AK (2013). The SLC26 gene family of anion transporters and channels. Mol Aspects Med.

[4] Ambort D, Johansson ME, Gustafsson JK, Ermund A, Hansson GC (2012). Perspectives on mucus properties and formation–lessons from the biochemical world. Cold Spring Harb Perspect Med.

[5] Aritake H, Tamada T, Murakami K, Gamo S, Nara M, Kazama I, Ichinose M, Sugiura H (2021). Effects of indacaterol on the LPS-evoked changes in fluid secretion rate and pH in swine tracheal membrane. Pflugers Arch.

[6] Ballard ST, Schepens SM, Falcone JC, Meininger GA, Taylor AE (1992). Regional bioelectric properties of porcine airway epithelium. J Appl Physiol.

[7] Bakouh N, Bienvenu T, Thomas A, Ehrenfeld J, Liote H, Roussel D, Duquesnoy P, Farman N, Viel M, Cherif-Zahar B, Amselem S, Taam RA, Edelman A, Planelles G, Sermet-Gaudelus I (2013). Characterization of SLC26A9 in patients with CF-like lung disease. Hum Mutat.

[8] Ballard ST, Spadafora D (2007). Fluid secretion by submucosal glands of the tracheobronchial airways. Respir Physiol Neurobiol.

[9] Blouquit S, Morel H, Hinnrasky J, Naline E, Puchelle E, Chinet T (2002). Characterization of ion and fluid transport in human bronchioles. Am J Respir Cell Mol Biol.

[10] Blouquit-Laye S, Chinet T (2007). Ion and liquid transport across the bronchiolar epithelium. Respir Physiol Neurobiol.

[11] Coakley RD, Boucher RC (2001). Regulation and functional significance of airway surface liquid pH. JOP.

[12] Coakley RD, Grubb BR, Paradiso AM, Gatzy JT, Johnson LG, Kreda SM, O'Neal WK, Boucher RC (2003). Abnormal surface liquid pH regulation by cultured cystic fibrosis bronchial epithelium. Proc Natl Acad Sci USA.

[13] Cooper JL, Quinton PM, Ballard ST (2013). Mucociliary transport in porcine trachea: differential effects of inhibiting chloride and bicarbonate secretion. Am J Physiol Lung Cell Mol Physiol.

[14] Darrah RJ, Mitchell AL, Campanaro CK, Barbato ES, Litman P, Sattar A, Hodges CA, Drumm ML, Jacono FJ (2016). Early pulmonary disease manifestations in cystic fibrosis mice. J Cyst Fibros.

[15] Danahay H, Gosling M (2020). TMEM16A: an alternative approach to restoring airway anion secretion in cystic fibrosis?. Int J Mol Sci.

[16] Dittrich NP, Kummer W, Clauss WG, Fronius M (2015). Luminal acetylcholine does not affect the activity of the CFTR in tracheal epithelia of pigs. Int Immunopharmacol.

[17] Engelhardt JF, Yankaskas JR, Ernst SA, Yang Y, Marino CR, Boucher RC, Cohn JA, Wilson JM (1992). Submucosal glands are the predominant site of CFTR expression in the human bronchus. Nat Genet.

[18] Folkesson HG, Matthay MA, Frigeri A, Verkman AS (1996). Transepithelial water permeability in microperfused distal airways. Evidence for channel-mediated water transport. J Clin Invest.

[19] Fuster DG, Alexander RT (2014). Traditional and emerging roles for the SLC9 Na^+^/H^+^ exchangers. Pflugers Arch.

[20] Garnett JP, Hickman E, Tunkamnerdthai O, Cuthbert AW, Gray MA (2013). Protein phosphatase 1 coordinates CFTR-dependent airway epithelial HCO3- secretion by reciprocal regulation of apical and basolateral membrane Cl–HCO3- exchangers. Br J Pharmacol.

[21] Hollenhorst MI, Lips KS, Wolff M, Wess J, Gerbig S, Takats Z, Kummer W, Fronius M (2012). Luminal cholinergic signalling in airway lining fluid: a novel mechanism for activating chloride secretion via Ca^2+^-dependent Cl^-^ and K^+^ channels. Br J Pharmacol.

[22] Ishiguro H, Steward MC, Yamamoto A (2011). Microperfusion and micropuncture analysis of ductal secretion. Pancreapedia Exocrine Pancreas Knowl Base.

[23] Jacquot J, Puchelle E, Hinnrasky J, Fuchey C, Bettinger C, Spilmont C, Bonnet N, Dieterle A, Dreyer D, Pavirani A, Dalemans W (1993). Localization of the cystic fibrosis transmembrane conductance regulator in airway secretory glands. Eur Respir J.

[24] Kim D, Huang J, Billet A, Abu-Arish A, Goepp J, Matthes E, Tewfik MA, Frenkiel S, Hanrahan JW (2019). Pendrin mediates bicarbonate secretion and enhances cystic fibrosis transmembrane conductance regulator function in airway surface epithelia. Am J Respir Cell Mol Biol.

[25] Kim D, Kim J, Burghardt B, Best L, Steward MC (2014). Role of anion exchangers in Cl^−^ and HCO_3_^−^ secretion by the human airway epithelial cell line Calu-3. Am J Physiol Cell Physiol.

[26] Kreda SM, Mall M, Mengos A, Rochelle L, Yankaskas J, Riordan JR, Boucher RC (2005). Characterization of wild-type and deltaF508 cystic fibrosis transmembrane regulator in human respiratory epithelia. Mol Biol Cell.

[27] Kreindler JL, Peters KW, Frizzell RA, Bridges RJ (2006). Identification and membrane localization of electrogenic sodium bicarbonate cotransporters in Calu-3 cells. Biochim Biophys Acta.

[28] Krouse ME, Talbott JF, Lee MM, Joo NS, Wine JJ (2004). Acid and base secretion in the Calu-3 model of human serous cells. Am J Physiol Lung Cell Mol Physiol.

[29] Lérias J, Pinto M, Benedetto R, Schreiber R, Amaral M, Aureli M, Kunzelmann K (2018). Compartmentalized crosstalk of CFTR and TMEM16A (ANO1) through EPAC1 and ADCY1. Cell Signal.

[30] Lee RJ, Foskett JK (2010). Mechanisms of Ca^2+^-stimulated fluid secretion by porcine bronchial submucosal gland serous acinar cells. Am J Physiol Lung Cell Mol Physiol.

[31] Lohi H, Kujala M, Makela S, Lehtonen E, Kestila M, Saarialho-Kere U, Markovich D, Kere J (2002). Functional characterization of three novel tissue-specific anion exchangers SLC26A7, -A8, and -A9. J Biol Chem.

[32] Mall M, Bleich M, Greger R, Schreiber R, Kunzelmann K (1998). The amiloride-inhibitable Na^+^ conductance is reduced by the cystic fibrosis transmembrane conductance regulator in normal but not in cystic fibrosis airways. J Clin Invest.

[33] Mall M, Boucher RC, Bush A, Alton EWFW, Davies JC, Griesenbach U, Jaffe A (2006). Pathogenesis of pulmonary disease in cystic fibrosis. Cystic fibrosis in the 21st century.

[34] McShane D, Davies JC, Davies MG, Bush A, Geddes DM, Alton EW (2003). Airway surface pH in subjects with cystic fibrosis. Eur Respir J.

[35] Meyerholz DK, Suarez CJ, Dintzis SM, Frevert CW, Treuting PM, Dintzis SM, Montine KS (2018). Respiratory system. Comparative anatomy and histology, a mouse, rat, and human atlas.

[36] Paradiso AM (1997). ATP-activated basolateral Na+/H+ exchange in human normal and cystic fibrosis airway epithelium. Am J Physiol.

[37] Paradiso AM, Coakley RD, Boucher RC (2003). Polarized distribution of HCO_3_^-^ transport in human normal and cystic fibrosis nasal epithelia. J Physiol.

[38] Pezzulo AA, Tang XX, Hoegger MJ, Abou Alaiwa MH, Ramachandran S, Moninger TO, Karp PH, Wohlford-Lenane CL, Haagsman HP, van Eijk M, Bánfi B, Horswill AR, Stoltz DA, McCray PB, Welsh MJ, Zabner J (2012). Reduced airway surface pH impairs bacterial killing in the porcine cystic fibrosis lung. Nature.

[39] Quinton PM (2008). Cystic fibrosis: impaired bicarbonate secretion and mucoviscidosis. Lancet.

[40] Roos A, Boron WF (1981). Intracellular pH. Physiol Rev.

[41] Saint-Criq V, Gray MA (2017). Role of CFTR in epithelial physiology. Cell Mol Life Sci.

[42] Salinas D, Haggie PM, Thiagarajah JR, Song Y, Rosbe K, Finkbeiner WE, Nielson DW, Verkman AS (2005). Submucosal gland dysfunction as a primary defect in cystic fibrosis. FASEB J.

[43] Schultz A, Puvvadi R, Borisov SM, Shaw NC, Klimant I, Berry LJ, Montgomery ST, Nguyen T, Kreda SM, Kicic A, Noble PB, Button B, Stick SM (2017). Airway surface liquid pH is not acidic in children with cystic fibrosis. Nat Commun.

[44] Shamsuddin AK, Quinton PM (2012). Surface fluid absorption and secretion in small airways. J Physiol.

[45] Shamsuddin AKM, Quinton PM (2019). Concurrent absorption and secretion of airway surface liquids and bicarbonate secretion in human bronchioles. Am J Physiol Lung Cell Mol Physiol.

[46] Sheppard MN, Hodson ME, Geddes DM (1995). The pathology of cystic fibrosis. Cystic fibrosis.

[47] Smith JJ, Travis SM, Greenberg EP, Welsh MJ (1996). Cystic fibrosis airway epithelia fail to kill bacteria because of abnormal airway surface fluid. Cell.

[48] Thomas JA, Buchsbaum RN, Zimniak A, Racker E (1979). Intracellular pH measurements in Ehrlich ascites tumor cells utilizing spectroscopic probes generated in situ. Biochemistry.

[49] Urbach V, Hélix N, Renaudon B, Harvey BJ (2002). Cellular mechanisms for apical ATP effects on intracellular pH in human bronchial epithelium. J Physiol.

[50] Weibel ER (1963). Morphometry of the Human Lung.

[51] Xu H, Ghishan FK, Kiela PR (2018). SLC9 gene family: function, expression, and regulation. Compr Physiol.

[52] Zajac M, Dreano E, Edwards A, Planelles G, Sermet-Gaudelus I (2021). Airway surface liquid pH regulation in airway epithelium: current understandings and gaps in knowledge. Int J Mol Sci.

[53] Zeiher BG, Eichwald E, Zabner J, Smith JJ, Puga AP, McCray PB, Capecchi MR, Welsh MJ, Thomas KR (1995). A mouse model for the delta F508 allele of cystic fibrosis. J Clin Invest.

